# Sericin Improves Cognitive Outcomes in a Mouse Model of Cerebral Ischemia: The Role of Blood-Brain Barrier Disruption and Edema

**DOI:** 10.34172/apb.46668

**Published:** 2026-06-28

**Authors:** Leila Hosseini, Seyed Mehdi Vatandoust, Narmin Farazi, Amir Pasokh, Saeed Sadigh-Eteghad, Mohammad Farzipour, Fereshteh Farajdokht, Alireza Mohajjel Nayebi, Ali Hassanzadeh, Javad Mahmoudi

**Affiliations:** ^1^Research Center of Psychiatry and Behavioral Sciences, Tabriz University of Medical Sciences, Tabriz, Iran; ^2^Neurosciences Research Center, Tabriz University of Medical Sciences, Tabriz, Iran; ^3^Department of Pharmaceutical Sciences, Division of Pharmacology and Toxicology, University of Vienna, Vienna, Austria; ^4^Vienna Doctoral School of Pharmaceutical, Nutritional and Sport Sciences, University of Vienna, Josef-Holaubek-Platz 2, 1090, Vienna, Austria; ^5^Physical Medicine and Rehabilitation Research Center, Tabriz University of Medical Sciences, Tabriz, Iran; ^6^Department of Pharmacology and Toxicology, Faculty of Pharmacy, Tabriz University of Medical Sciences, Tabriz, Iran; ^7^Department of Applied Cell Sciences, School of Advanced Technologies in Medicine, Tehran University of Medical Sciences, Tehran, Iran; ^8^Medical Philosophy and History Research Center, Tabriz University of Medical Sciences, Tabriz, Iran

**Keywords:** Brain edema, Sericin, Blood-brain barrier, Oxidative stress, Photothrombotic stroke, Memory

## Abstract

**Purpose:**

Oxidative stress, edema, and blood-brain barrier (BBB) dysfunction are involved in the pathophysiology of stroke and may offer opportunities for managing its related insults. Sericin (Ser) has neuroprotective and pro-cognitive effects. This study aimed to determine whether Ser could reduce cerebral edema and its associated molecular and cognitive abnormalities in a mouse model of cortical ischemia.

**Methods:**

The mice were allocated into five cohorts: Sham, normal saline (NS), Ser200, Ser300, and Ser400. Each group received its treatment for 14 days before being subjected to cerebral ischemia or a sham surgery procedure. Spatial memory was assessed using the Lashley III maze. Brain edema, BBB permeability, malondialdehyde (MDA) level, and changes in anti-oxidant markers (total antioxidant capacity (TAC), as well as superoxide dismutase (SOD) and glutathione peroxidase (GPx) activities) were evaluated in the brain samples. Additionally, the expression of BBB structural integrity proteins, including occludin and zonula occludens-1 (ZO-1), and matrix metalloproteinase-9 (MMP-9), were analyzed.

**Results:**

Ser300 and Ser400 improved spatial memory (at least *P*<0.05). Also, Ser400 reduced brain water content (*P*<0.05), Ser300 and 400 decreased BBB leakage (at least *P*<0.05), and normalized anti-oxidant markers (at least *P*<0.05). As well, Ser400 diminished MDA content (*P*<0.05). All doses decreased MMP-9 levels (at least *P*<0.01). Ser400 upregulated the expression of occludin (*P*<0.01), and Ser300 and 400 increased ZO-1 proteins (at least *P*<0.05).

**Conclusion:**

Ser improved cognitive dysfunction associated with BBB damage and edema induced by cerebral ischemia in a mouse model.

## Introduction

 Among various mechanisms contributing to ischemic stroke, oxidative stress has a twofold deleterious effect on stroke outcomes.^1^ On the one hand, it exacerbates behavioural impairments leading to permanent motor and cognitive disabilities; on the other side, it disrupts cells (i.e., microvascular endothelial cells, pericytes, and astrocytes) and machineries (i.e., matrix metalloproteinases (MMPs), tight junctions (TJs), and basement membranes) involved in blood-brain barrier (BBB) integrity.^2-4^ Importantly, activation of MMP-9 reduces the expression levels of TJ proteins^5^ and degrades basement membranes, which culminate in the breakdown of the BBB.^6,7^ The changes in BBB structure and function reduce its capacity to control the penetration of macromolecules and harmful substances into the cerebral parenchyma and preserve cerebral homeostasis.^8-10^ Consequently, following BBB disruption, blood components enter the cerebral parenchyma, eliciting oxidative stress that exacerbates BBB disintegration and contributes to further penetration of blood components, resulting in a vicious cycle that worsens vasogenic brain edema by promoting swollen tissues and increased intracranial pressure (ICP). Increased ICP exerts mechanical force on cerebral structures and capillaries, leading to hypoperfusion and increased infarct volume, and neuronal damage.^11,12^ Given the close relationship between oxidative stress and disruption of BBB integrity and subsequent brain edema in ischemic stroke^11^, the development of novel neuroprotective strategies with antioxidant and anti-edema potential is of great importance.

 Post-stroke cognitive deficits encompass a decrease in different cognitive domains, including learning, memory, and executive abilities, potentially affecting stroke survivors’ quality of life. ^13^ Numerous factors contributing to cognitive impairment following a stroke are investigated, including the stroke’s location, type, volume, severity, vascular risk factors like hypertension and diabetes, as well as underlying mechanisms.^14,15^ Key areas of importance include the dominant hemisphere and lesions affecting the prefrontal-subcortical circuit, both of which are crucial in mediating executive dysfunction.^15^ The functions of the frontal lobe, such as processing speed, working memory, reaction time, and performance on executive tasks, are most commonly affected following a stroke. A large, localized cortico-subcortical ischemic lesion in a region critical to cognitive function can result in a rapid and significant decline in cognitive abilities.^16^ Cognitive deficits following stroke arise from mechanisms such as changes in redox balance, mitochondrial impairment, BBB disruption, lymphatic system impairment, neuroinflammation, and amyloid-β accumulation in the brain parenchyma.^14,17^

 Sericin (Ser) is a natural, hydrophilic protein derived from the cocoon of the silkworm *Bombyx mori *(Bombycidae), constituting approximately 30% of the total weight of the cocoon.^18^ Composed of 18 amino acids, it is particularly rich in hydrophilic essential amino acids, such as serine and glycine^19^ and its physicochemical properties, including hydrophilicity, water solubility, biodegradability, and biocompatibility, make it a versatile compound for biomedical purposes.^20^ (Figure 1) Its ability to improve cognitive function and exhibit antidepressant and anxiolytic properties, partly through its antioxidant, anti-inflammatory, anti-apoptotic, and regenerative properties, makes it an ideal neuroprotective compound for neurological conditions.^21,22^ Recent findings have demonstrated that Ser enhances spatial learning and memory, elevates the activity of antioxidant enzymes, and attenuates apoptosis along with inflammation in the hippocampus following ischemic brain injury^23^, indicating its neuroprotective potential for cerebral ischemia. However, whether Ser reduces cognitive impairments via minimizing BBB disintegration under ischemic stroke remains still unknown. Therefore, this study aims to examine the effects of this peptide on cognitive outcomes, brain edema, oxidative stress, and BBB integrity markers in a mouse model of focal cerebral ischemia.

**Figure 1:**
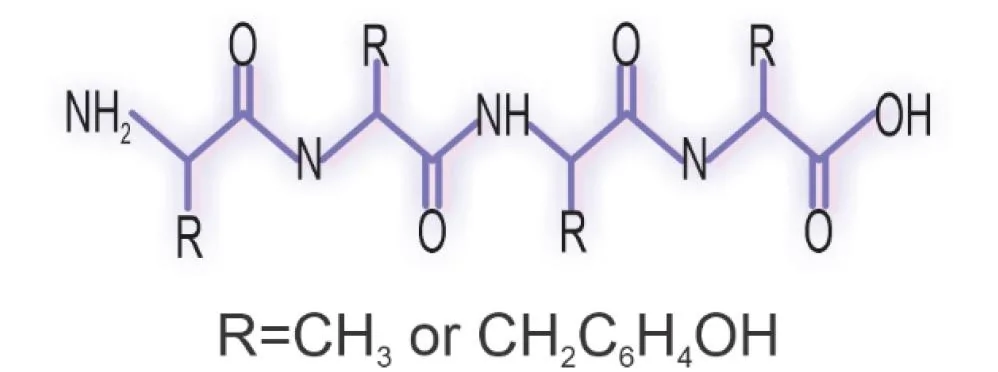
Biochemical structure of silk sericin

## Material and Methods

###  Animals

 Adult male Balb/c mice, each weighing between 25 and 30 g, were purchased from Homa Teb Tabriz Company (HTT Co., Tabriz, Iran). The mice were housed in standard polypropylene cages, with a density of five mice per cage, under a controlled 12:12 light/dark cycle and maintained at a temperature of 25 ± 2°C, and standard light exposure. Mice had access to drinking water and a standard rodent diet. All experimental protocols adhered to the National Institutes of Health (NIH) guidelines for the care and use of laboratory animals (Publication No. 85–23, revised 1985).

###  Study Design

 After acclimatization for seven days, the mice were randomly allocated to the following five groups (n = 19/group): Sham, normal saline (NS), Ser200, Ser300, and Ser400. In all groups, mice received their respective treatment for 14 consecutive days. On the final day of treatment (day 14), all mice underwent a photothrombosis procedure to induce focal cerebral ischemia, except for the sham group, which was subjected only to sham surgery. Mice in both the sham and NS groups were pretreated with NS. The Ser200, Ser300, and Ser400 groups received 200, 300, and 400 mg/kg of Ser (Xian Lyphar Biotech Co., Xi’an, China). All solutions were given via the oral route (gavage) at a constant volume of 10 ml/kg. Experiments in each group were conducted on three sets of mice: the first set was used to study changes in BBB permeability (n = 6), the second set was used for estimation of brain edema (n = 4), and the third set (n = 9) was used for behavioural and biochemical assays. At the end of the behavioural tests, six mice were used to assess oxidative stress parameters, and three mice were allocated for Western blot analysis (Figure 2).

**Figure 2:**
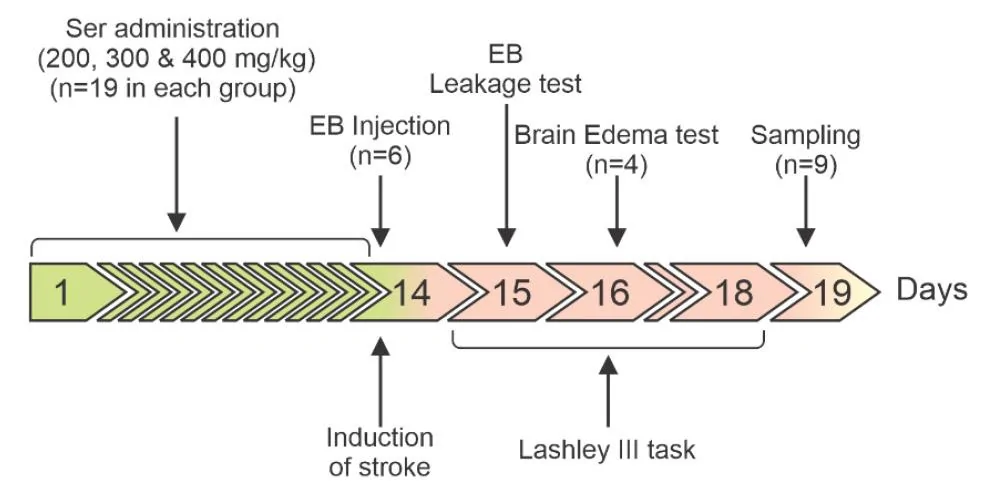
The timescale of the study

###  Photothrombosis Procedure

 Focal cerebral ischemia was induced using the photothrombotic model. Mice were anesthetized with isoflurane (4% induction and 2% maintenance) delivered in an oxygen mixture. Anesthetized mice were fixed in a stereotaxic apparatus, and a midline scalp incision was performed to expose the skull. Then, Rose Bengal dye (150 µg/g; Sigma-Aldrich, St. Louis, Missouri, USA) was slowly injected intraperitoneally (i.p.) 2 min before light exposure. A green laser (70 mW, 2 mm beam diameter) was then directed at the medial prefrontal cortex (mPFC) of the right hemisphere (anterior-posterior: 2.0; medial-lateral: +0.5 relative to the bregma) for 10 min to initiate localized photochemical thrombosis.^24^ Upon completion of the procedure, the incision site was sutured, and the animal was returned to its cage for a 24 h recovery period. The sham mice group was subjected to the same procedure, except that they received only NS as the Rose Bengal dye vehicle.

###  Lashley III Maze

 The assessment of learning and spatial reference memory was performed using the Lashley III maze task over four consecutive days as reported previously, with a minor modification.^25^ The maze was made of black Plexiglas with lateral walls and a transparent ceiling. It consisted of a start box (8 × 9.5 × 7 cm) with a 4 × 4 cm guillotine door, four connected lanes (45 cm long, 7 cm high, and 5 cm wide), and a goal box (8 × 9.5 × 7 cm). A piece of food reward was placed in the goal box before the task. The food-deprived mouse was positioned in the start box of the maze and kept there for 15 s. The guillotine door was then lifted, allowing the mouse to explore the maze and reach the goal box. Before each trial, the maze was thoroughly cleaned with a 70% ethanol solution to eliminate any scent cues.

###  Sampling

 For all sampling procedures, mice were anesthetized with a ketamine and xylazine mixture (100 mg/kg and 10 mg/kg, i.p.) and underwent a rapid brain extraction technique, originally developed for rats and adapted with minor modifications for mice, to remove the brain from the skull.^26^

###  Measurement of BBB Permeability

 Evans blue solution (4% in saline, 4 mL/kg; Sigma-Aldrich, St. Louis, Missouri, United States) was injected via the tail vein into six mice approximately 1 minute after photothrombosis, and brain samples were collected 24 hours later. On the sampling day, following transcranial perfusion with heparinized normal saline (10 U/mL in physiological saline), the extracted brains were divided into lesioned and intact hemispheres using a mouse brain matrix (Arman Poshtiban Teb, Tabriz, Iran). Each hemisphere was then homogenized in 1 mL PBS containing 50% trichloroacetic acid, and the samples were centrifuged to measure the absorbance of Evans blue in the supernatants by spectrophotometry at 620 nm. The Evans blue leakage index was calculated as the ratio of absorbance in the lesioned hemisphere to that in the intact hemisphere.^27,28^

###  Assessment of Brain Edema

 Forty-eight hours after photothrombosis, the extracted brains (n = 4/group) were placed in a brain matrix. After isolation of the cerebellum and olfactory bulb, the hemispheres were separated from each other along the interhemispheric plane. The wet weight (WW) of each hemisphere was measured separately. To obtain the dry weight (DW), the samples were dried in a 110°C oven for 24 h. Finally, the percentage of brain water content was calculated according to the formula: (WW−DW)/WW × 100.^29^ The selection of the mentioned time point for edema assessment was based on evidence indicating that brain edema reaches its peak forty-eight hours after photothrombosis in mice.^30,31^

###  Measurement of Oxidative Stress Markers

 For assessing oxidative stress markers, the enzymatic activities of glutathione peroxidase (GPx) and superoxide dismutase (SOD), and also the concentration of malondialdehyde (MDA), and the level of total antioxidant capacity (TAC) were measured in brain samples (n = 6/group). Briefly, samples were homogenized in a 1.15% KCl solution and centrifuged at 1000 rpm at 4°C for 10 min.

 Both SOD and GPx activities were measured spectrophotometrically using the RANSOD and Ransel kits (Randox Laboratories Ltd, Crumlin, UK) at 37°C, respectively at 505 nm and 340 nm, and results were reported as units (U)/mg protein. TAC was assessed using the Randox total antioxidant status assay kit (Randox Laboratories Ltd, Crumlin, UK) by measuring the reduction of ferric ion to ferrous ion at 600 nm, and results were expressed as µmol/mg of protein. MDA levels were determined using the TBARS assay (Thiobarbituric Acid Reactive Substances assay), which measures MDA as an indicator of lipid peroxidation at 535 nm and expressed as nmol/mg protein.^32,33^

###  Assessment of Protein Expression

 To analyze the expression of MMP-9, zonula occludens-1 (ZO-1), and occludin proteins by western blot, samples (n = 3/group) were homogenized in ice-cold radioimmunoprecipitation assay (RIPA) buffer supplemented with a protease inhibitor cocktail. Protein content was measured using Bradford’s method. Protein samples were then separated by electrophoresis on a 12.5% sodium dodecyl sulfate-polyacrylamide gel (SDS-PAGE) and transferred onto polyvinylidene difluoride (PVDF) membranes (Bio-Rad, Hercules, California, USA). The membranes were then blocked in PBS containing 5% non-fat dry milk and 0.05% Tween-20 for 60 min at room temperature. After washing the membranes for 5 min in wash buffer, they were incubated with primary antibodies against MMP-9 (ab283575), ZO-1 (ab190085), occludin (ab216327), and β-actin (ab8227), all from Abcam, Cambridge, UK. The same membranes were re-probed with different antibodies. Afterward, the membranes were washed three times with TBST and incubated with the secondary antibody. Immunoreactive bands were visualized by using an enhanced chemiluminescence kit. Image J software was employed to determine the blot density. The internal control β-actin was used for normalization of the density values of the blots.^34,35^

###  Statistical Analysis

 Data are presented as the mean ± SEM and were analyzed with GraphPad Prism 10 (GraphPad Software Inc., La Jolla, CA, USA). Data were analyzed using a two-way ANOVA for the Lashley III maze or a one-way ANOVA, followed by Tukey’s post hoc test. A *p*-value less than 0.05 was considered statistically significant.

## Results

###  Lashley III Maze

 The results of a two-way ANOVA for latency time revealed significant effects of group (F (4, 140) = 20.9, *P* < 0.001), day (F (3, 140) = 412, *P* < 0.001), and group × day interaction (F (12, 140) = 2.44, *P* = 0.0066). In comparison to the sham group, the NS group exhibited a notably longer latency in finding the goal box on days 3 and 4 (*P* < 0.001). However, a notable reduction in latency was observed in the Ser300 group (*P* < 0.01 on day 3) and the Ser400 group (at least *P* < 0.05 on days 3 and 4) (Figure 3A).

**Figure 3:**
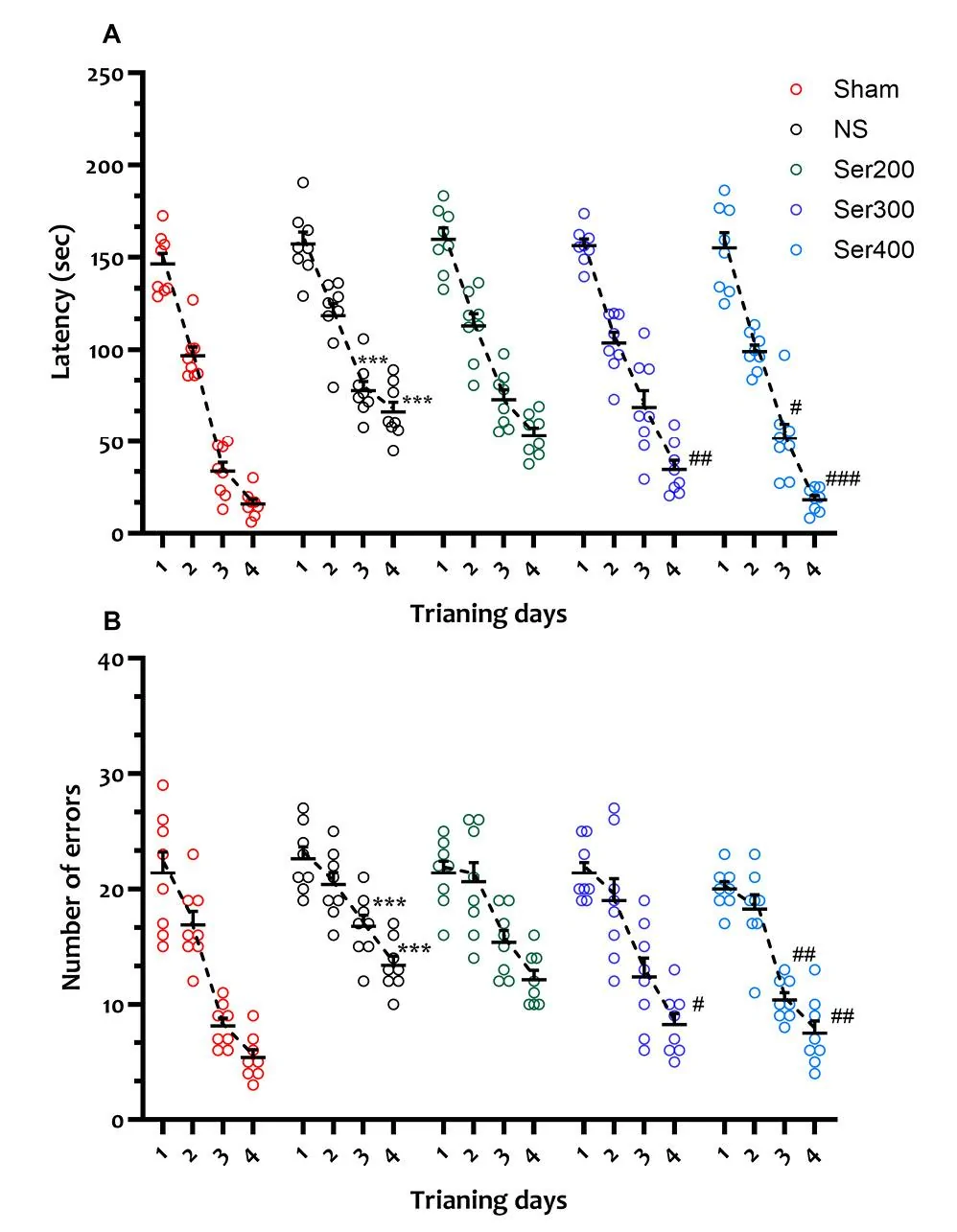
The effects of different doses of Ser on (A) latency and (B) number of errors in the Lashley III Maze task.Values are mean ± SEM. (n = 8/group). ^***^P < 0.001 vs. sham group, ^#^*P* < 0.05, ^##^*P* < 0.01, ^###^*P* < 0.001 vs. NS group. NS: normal saline; Ser: Sericin

 We also found significant effects of group (F (4, 140) = 15.29, *P* < 0.001) and day (F (3, 140) = 118.6, *P* < 0.001), but no significant group × day interaction (F (12, 140) = 1.738, *P* = 0.0647) for the number of errors. Focal ischemia significantly increased the number of errors in the NS group on days 3 and 4 versus the sham group (*P* < 0.001). Nevertheless, the number of errors was significantly reduced in the Ser300 group (*P* < 0.05 on day 4) and the Ser400 group compared to the NS group (*P* < 0.01 on days 3 and 4) (Figure 3B).

###  Oxidative Stress

 The one-way ANOVA proved a marked effect of the Ser on MDA level (F (4, 25) = 5.938, *P* = 0.0017), TAC (F (4, 25) = 14.22, *P* < 0.0001) as well as SOD (F (4, 25) = 11. 43, *P* < 0.0001) and GPx (F (4, 25) = 15.09, *P* < 0.0001), enzymatic activities.

 Focal ischemia led to a marked increase in MDA levels (*P* < 0.01, Figure 4A), while it markedly declined TAC (*P* < 0.001, Figure 4B) as well as the enzymatic activities of SOD (*P* < 0.001, Figure 3C) and GPx (*P* < 0.001, Figure 4D) compared to the NS group. Pretreatment with Ser (400 mg/kg) significantly reduced MDA levels (*P* < 0.05). Moreover, Ser at 300 and 400 mg/kg significantly increased TAC and enhanced the activities of both SOD and GPx, with each dose showing at least *P* < 0.05 for TAC and both enzymes versus the NS group.

**Figure 4:**
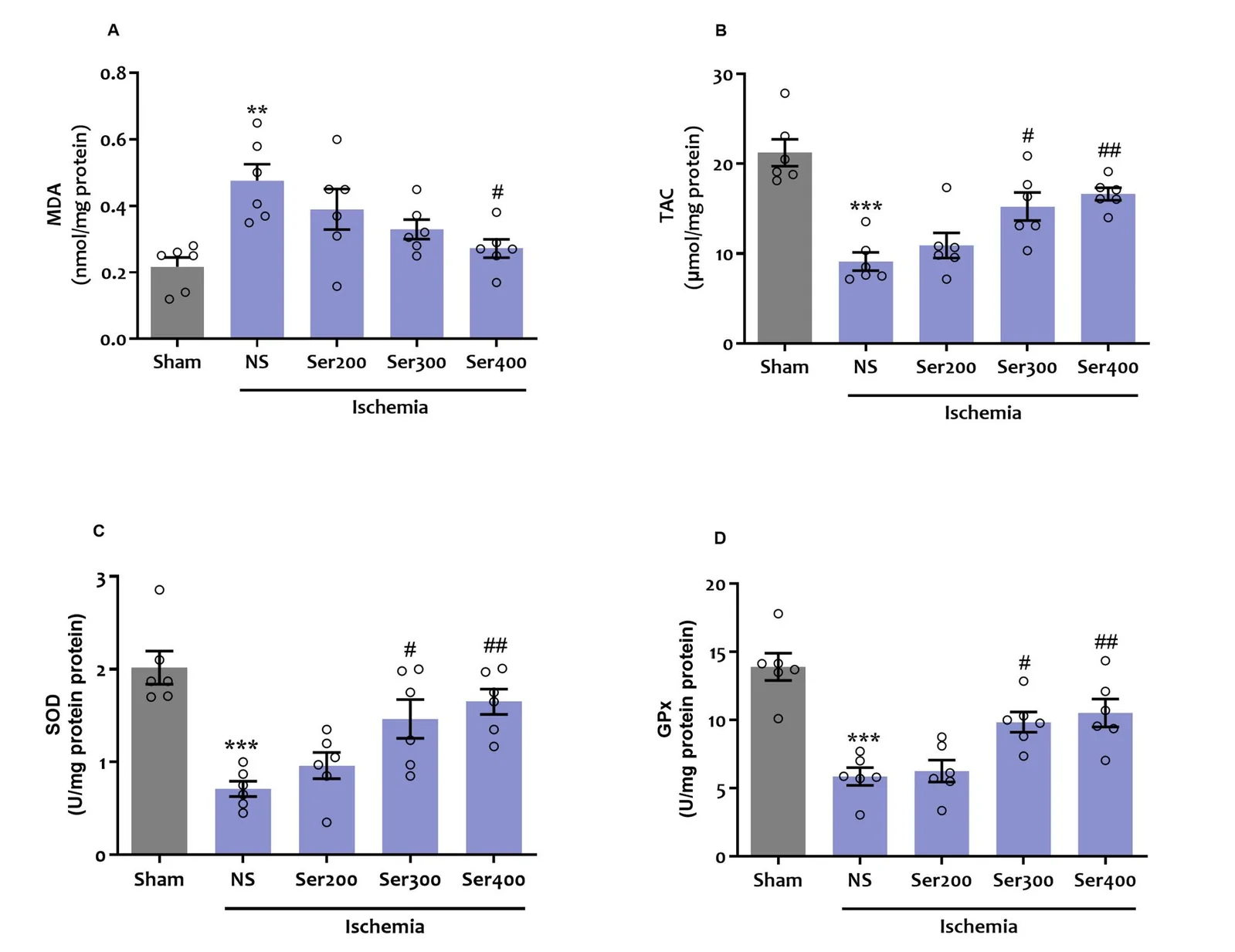
Effects of different doses of Ser on (A) MDA levels, (B) TAC, as well as (C) SOD and (D)GPx enzymatic activities.All values are mean ± SEM (n = 6/ group). ^**^P < 0.01 and ^***^P < 0.001 vs. sham group. ^#^*P* < 0.05, ^##^*P* < 0.01 vs. NS group. NS: normal saline; Ser: Sericin; MDA: malondialdehyde; TAC: total antioxidant capacity; SOD: superoxide dismutase; GPx: glutathione peroxidase

###  Evans Blue Leakage Index and Edema

 As depicted in Figure 5A, a statistically significant difference for the Evans blue leakage index was detected among the study groups (F (4, 25) = 56.44, *P* < 0.0001). Induction of focal ischemia significantly increased the Evans blue leakage index in NS mice in comparison to sham mice (*P* < 0.001). On the other hand, pretreatment with Ser at doses of 300 and 400 mg/kg reduced this index in mice with focal cerebral ischemia (at least *P* < 0.05).

**Figure 5:**
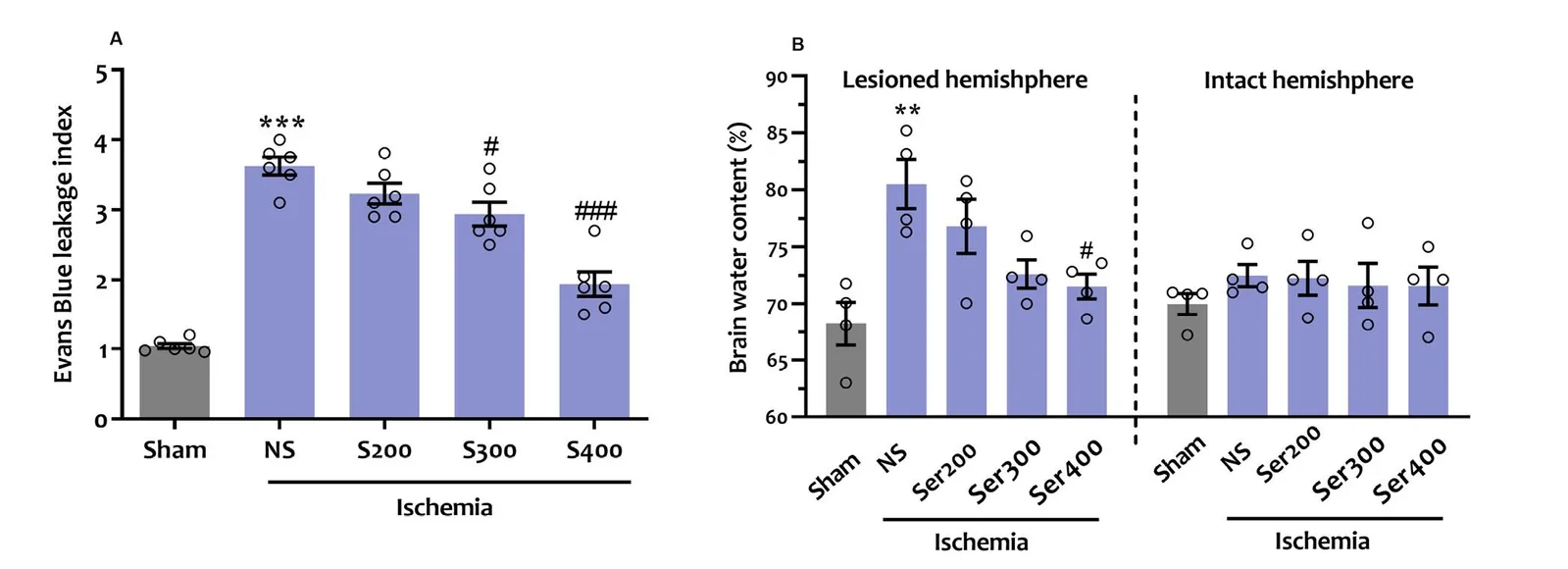
The effects of different doses of Ser on (A) Evans blue leakage index and (B) brain water content. All values are mean ± SEM (n = 6/ group for Evans blue extraversion and n = 4/ group for brain water content). ^**^P < 0.01 and ^***^ P < 0.001 vs. sham group. ^#^*P* < 0.05, ^###^*P* < 0.001 vs. the NS group. NS: normal saline; Ser: Sericin

 The results of one-way ANOVA for the percentage of brain water content showed a significant difference among groups in the lesioned hemisphere (F (4, 15) = 6.848, *P* = 0.0024), but not in the intact hemisphere (F (4, 15) = 0.4493, *P* = 0.7714). As shown in Figure 5B, there was a significant increase in the percentage of brain water content (*P* < 0.01) in the NS group versus the sham group. Nevertheless, this parameter displayed a significant decrease in Ser400 group (*P* < 0.05), indicating the effectiveness of this regimen for the modulation of brain edema.

###  MMP-9, Occludin, and ZO-1 Levels

 The one-way ANOVA showed significant differences in the expression of MMP-9 (F (4, 10) = 42.1, *P* < 0.0001), occludin (F (4, 10) = 21.87, *P* < 0.0001), and ZO-1 (F (4, 10) = 23.35, *P* < 0.0001) among the study groups. As depicted in Figure 6A, the MMP-9 was upregulated in the NS group compared to the sham group (*P* < 0.001), while all doses of Ser could significantly decrease the levels of MMP-9 compared to the NS group (at least *P* < 0.01). Ischemic stroke noticeably reduced the protein levels of occludin and ZO-1 (*P* < 0.001) compared with the mice in the sham group. Interestingly, Ser pretreatment at the dose of 400 mg/kg could significantly reverse the suppressed levels of occludin (*P* < 0.01, Figure 6B). In addition, Ser at doses of 300 and 400 mg/kg meaningfully increased ZO-1 level (at least *P* < 0.05) in comparison with the NS group (Figure 6C).

**Figure 6:**
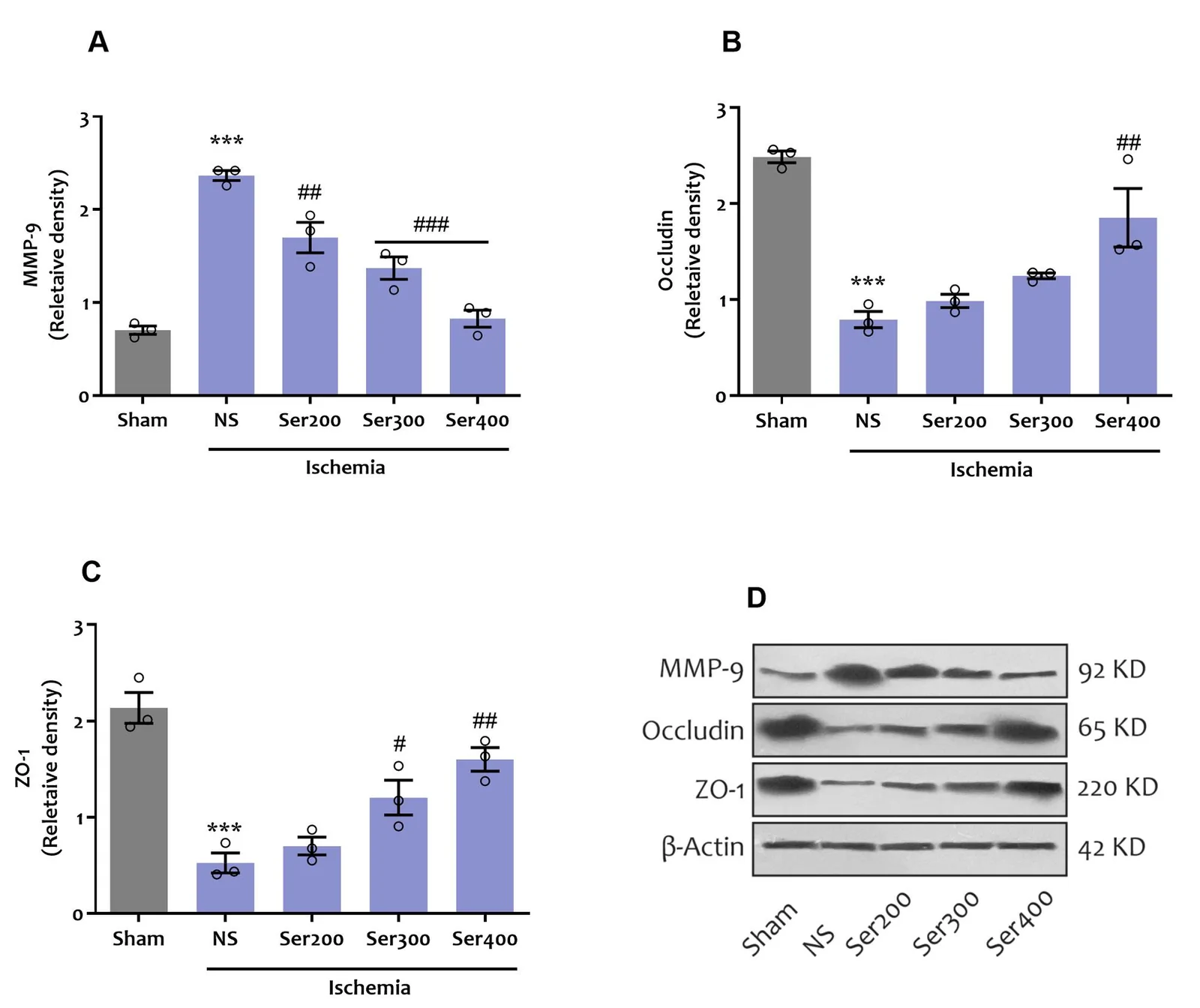
Effects of different doses of Ser on the expression of (A) MMP-9, (B) occludin, (C) ZO-1, and (D) representative blot images. All values are expressed as mean ± SEM (n = 3). ^***^P < 0.001 versus the sham group. ^#^*P* < 0.05, ^##^*P* < 0.01, and ^###^*P* < 0.01 versus the NS group. NS: Normal saline; Ser: Sericin

## Discussion

 The current study demonstrated that pretreatment with Ser exerted neuroprotective effects against ischemic injury, in part by preventing BBB disruption, edema, increased oxidative stress burden, and MMP-9 elevation, as well as by normalizing ZO-1 and occludin expression.

 At behavioral levels, induction of focal cortical ischemia resulted in deficits in spatial memory and learning, as indicated by a prominent increase in task completion latency and committed errors for accessing the goal box in the Lashlay III maze. However, pretreatment with Ser significantly improved cognitive function following ischemic damage. Previous studies have shown that ischemia in the mPFC is associated with impairments across various cognitive domains, including social recognition, episodic-like, and spatial memories.^24,36,37^ Given the critical role of the mPFC and hippocampus interaction in integrating different types of memory, damage to the mPFC, even in the presence of an intact hippocampus, can result in cognitive dysfunctions.^24^ However, while abnormalities in sensorimotor performances appear to be recoverable in ischemic animals, the cognitive impairments following cortical ischemia tend to be more persistent. These impairments likely arise from distinct yet functionally interrelated mechanisms, such as mitochondrial and synaptic protein dysfunctions, BBB disruption leading to vasogenic edema, and oxidative stress.^37,38^ In agreement with our finding related to the pro-cognitive effect of Ser, previous reports from our laboratory and other researchers exhibited Ser’s potential for improving learning and memory deficits in different experimental models.^33,39-41^

 Additionally, our results indicated that ischemic injury elevated MDA levels and decreased TAC as well as SOD and GPx activities, all of which reflect a reduced neuronal capacity to neutralize reactive molecules generated by the ischemic insults. However, pretreatment with Ser (300 and 400 mg/kg) effectively restored antioxidant status by enhancing GPx and SOD activities, increasing TAC, and lowering MDA levels. Oxidative stress, which appears to be one of the most prominent pathological factors in stroke, affects neuronal survival by damaging key macromolecules (i.e., DNA, proteins, and lipids). It triggers various cell signaling pathways that can ultimately lead to edema, BBB dysfunction, endothelial dysfunction, irreversible neuronal injury, and memory deficits associated with ischemic stroke.^6,37,42,43^ Evidence from experimental models of cerebral ischemia reveals that antioxidant regimens can attenuate neuronal injuries and consequent cognitive impairments, primarily by reducing neuronal cell death, BBB disruption, and inflammation.^36,37,42^

 The ability of Ser to inhibit lipid peroxidation and stabilize free radicals, as demonstrated in in vitro experiments^44,45^, highlights its potential to reduce oxidative stress and protect neuronal structures.^23,39,41,46^ One of the mechanisms by which Ser mediates its antioxidant activity is attributed to the high content of hydroxyl amino acids, such as serine and threonine. The hydroxyl groups in these amino acids provide reducing power and enable trace element chelation.^45^ Moreover, mechanisms such as the inhibition of tyrosinase activity and the enhancement of antioxidant enzyme activities ^23,33^ further elucidate the antioxidant function of Ser.

 Our results demonstrated a significant increase in BBB permeability following ischemia. This event was accompanied by the development of brain edema. Pretreatment with Ser markedly reduced BBB permeability and significantly mitigated edema formation. These findings suggest that Ser may have a protective role in preserving BBB function during ischemic insults. Cerebral edema is characterized by the accumulation of fluid in the brain, leading to increased ICP, which impairs cerebral perfusion and results in neuronal injury, cognitive dysfunction, and, in severe cases, even death.^47,48^

 In agreement with previous findings, our western blotting assay demonstrated a significant increase in MMP-9 levels following cerebral ischemic injury, consistent with the previous findings.^49,50^ There is a well-established link between oxidative stress and MMP-9 expression in ischemic stroke and subsequent BBB damage.^51^ Additionally, oxidative stress increases neuronal susceptibility to ischemia-reperfusion injury and triggers numerous molecular cascades mediating MMP-9 activation.^52^ MMPs are a class of zinc-dependent proteolytic enzymes that play a crucial role in regulating various biological functions. MMP-9 is involved in several key processes, including morphogenesis, wound healing, and neurite outgrowth in the central nervous system.^53^ Of the 23 identified MMPs, MMP-2 and MMP-9 are the most extensively studied in the context of stroke due to their pivotal contribution to the pathogenesis of BBB disruption and the resulting vasogenic edema.^54^ It has been indicated that after brain ischemia, both expression and activity of the MMP-9 are markedly upregulated within 12-48 h, which is related to degrading extracellular matrix and intercellular TJs, leading to increased permeability of the BBB and brain edema formation.^55^ According to former studies, inhibiting MMP-9 can diminish the permeability of the BBB and alleviate brain injury.^56^ Moreover, activation of MMP-9 is a critical factor in the pathophysiology of various neuronal disorders, including ischemic stroke, meningitis, and encephalitis.^57-59^ As our results showed, Ser reduces the levels of MMP-9 compared to the NS group. In line with this, molecular docking analysis demonstrated that Ser could inhibit both MMP-9 and MMP-2, with binding energies of -6.5 kcal/mol and -5.4 kcal/mol, respectively. This indicates its efficient interaction with these enzymes and its potential for therapeutic use in stroke-related neuroprotection.^60^ We also found that induction of ischemic stroke downregulated the expression levels of occludin and ZO-1, whereas pretreatment with Ser normalized the expression of these components in the brain endothelium.

 Occludin is also important for neovascularization and angiogenesis, and may also have a function for endothelial cell proliferation, sprouting, and tube formation.^61^ ZO-1 plays a critical role in cell polarity, cytoskeleton organization^62^, TJs localization ^63^, closely influencing BBB permeability.^64^ The interaction between occludin and ZO-1 has been structurally characterized and is thought to influence occludin localization at TJs.^65,66^ Indeed, the architecture of the BBB owes to the coordinated interaction of TJs proteins, including occludin, ZO-1, and claudin-5, which orchestrate the maintenance of brain homeostasis by regulating cerebral vessel permeability and controlling molecular exchange between the blood and cerebral tissue.^67^ Under ischemic conditions, TJs proteins such as ZO-1 and occludin are downregulated through enzymatic degradation by MMPs and caspases, as well as redistribution or internalization. This disruption compromises TJs’ integrity and promotes BBB leakage. The resulting damage facilitates oxidative stress and inflammatory responses, which in turn further suppress and degrade TJs proteins, creating a self-reinforcing cycle. Ultimately, this cascade exacerbates cerebral edema and worsens neurological deficits.^67-70^

 As limitations, this study only investigated Ser as a pretreatment, which restricted the findings regarding its full therapeutic potential. Additional research is therefore needed to examine the efficacy of Ser when administered at different time windows after stroke induction, including both early and delayed treatment paradigms. Furthermore, detailed mechanistic investigations are needed to clarify the molecular pathways through which Ser exerts its neuroprotective effects, including its impact on other relevant signaling cascades involved in BBB integrity and neuronal survival.

 Also, female mice were excluded from the present study in order to minimize the potential influence of hormonal fluctuations associated with the estrous cycle, which may affect behavioral, biochemical, and molecular outcomes and increase variability in the data. However, inclusion of female gender and comparison of neuroprotective effect of sericin between both sexes would provide valuable information regarding possible sex-dependent effects.

## Conclusion

 The findings of this study indicated that pretreatment with Ser protected BBB integrity against acute ischemic stroke, reduced vasogenic brain edema, and improved spatial memory. These beneficial effects on BBB function, brain edema, and cognition appeared to result from Ser’s antioxidant properties and its inhibitory effects on MMP-9; however, further studies are needed to elucidate Ser’s neuroprotective effects under ischemic conditions.

## Competing Interests

 There are no conflicts of interest.

## Data Availability Statement

 The team of authors is committed to ensuring that the data supporting our findings in this research will be made available to interested parties upon reasonable request. The requested data are available by contacting the corresponding authors.

## Ethical Approval

 The study received approval from the Ethics Committee of Tabriz University of Medical Sciences (Approval code: IR.TBZMED.VCR.REC.1400.191).
